# Association between hypocalcemia and rivaroxaban in coagulation disorders: a case report

**DOI:** 10.31744/einstein_journal/2020RC4819

**Published:** 2020-01-22

**Authors:** Alexandre Mio Pos, Mateus Arruda Aleixo, Ana Paula Drummond-Lage

**Affiliations:** 1 Faculdade Ciências Médicas de Minas Gerais Belo HorizonteMG Brazil Faculdade Ciências Médicas de Minas Gerais, Belo Horizonte, MG, Brazil.

**Keywords:** Rivaroxaban/adverse effects, Blood coagulation, Hematoma, Parathyroidectomy

## Abstract

We describe a patient with tertiary hyperparathyroidism with history of three episodes of deep vein thrombosis and on rivaroxaban. The patient underwent a subtotal parathyroidectomy, developing cervical hematoma with airway compression. Therefore, emergency surgical decompression was necessary. Later, on the ninth postoperative day, the serum ionized calcium levels were low. Medical team knowledge about preexisting diseases and their implication in the coagulation state are essential conditions to reduce morbidity and mortality of surgeries. However, no reports were found in literature about the association of hypocalcemia with the use of the new class of anticoagulants, which act as factor X inhibitors (Stuart-Prower factor), predisposing to increased bleeding in the immediate postoperative period.

## INTRODUCTION

Rivaroxaban is an anticoagulant of the new class of selective inhibitors of activated factor X, being widely used for prevention and treatment of thromboembolic events, specifically in patients with cardiovascular diseases.^1^ The use of rivaroxaban use does not require coagulation monitoring, since this drug has predictable pharmacological properties, such as short half-life, and rapid action, with an improved safety profile which leads to a fixed dose use.^1^ However, in some potentially severe clinical situations, an analysis of the function of rivaroxaban should be conducted, even if indirectly, by measuring prothrombin time or determining activity of the anti-factor Xa.^1^ Among these clinical conditions, patients with chronic kidney disease stage 4 or 5, or with hyperparathyroidism stand out, because they comprise a group at high risk of thrombotic events. The use of anticoagulants use in this group of patients is not uncommon.

Serum levels of oral anticoagulants depend directly on renal function. Therefore, a reduction in renal clearance may affect the efficacy and safety of these drugs. Patients with chronic kidney disease have their own pharmacokinetic curve, above the normality threshold, which should be taken into consideration when analyzing the risk of hemorrhage.^1 , 2^ Due to these factors, clinical management of anticoagulant therapy in patients with chronic kidney disease has become a major medical challenge.

Written consent form was obtained from the patient.

## CASE REPORT

We present a 51-year-old male patient, with a history of chronic kidney disease stage 5, of uncertain etiology, on dialysis since 2003, with living-donor and HLA/ABO-incompatible renal transplant in 2014. As complications of the disease, the patient presented dyslipidemia, hyperuricemia, hypovitaminosis D (17ng/mL), osteoporosis with T-score -5.9, extra-skeletal calcification with advanced atherosclerosis, three thromboembolic events in inferior limb veins, and tertiary hyperparathyroidism. Continuous use of rivaroxaban 20mg per day and cinacalcet 30mg per day was initiated. Since the patient maintained intense osteoclastic activity with parathormone levels above 300pg per mL and ionized calcium constantly above the maximum limit of normality (1.60mg/dL) 2 years after the transplant, and with renal function already normalized (creatinine 1.10mg/dL), the team chose to perform partial excision of the parathyroids. Rivaroxaban was discontinued 48 hours before the surgical procedure, the anti-factor Xa was measured (0.004IU/mL). Next, the bridging anticoagulation program was initiated with low molecular weight heparin for 6 days. The procedure went with no abnormalities, and with laboratory criteria of effective excision (perioperative parathormone 8pg/mL). During intensive therapy care, the patient evolved with only asymptomatic hypocalcemia, controlled with oral calcium. On the fifth postoperative day, rivaroxaban 20mg per day was reinitiated, with no loading dose, followed by hospital discharge. The patient evolved well, however, quite late, on the nineth postoperative day, he presented ionized calcium (0.79mmol/L) despite the oral replacement of calcium (4.5g per day), calcitriol (2.25mg per day), and magnesium (3g per day), and sudden onset of incisional pain and discomfort, which were not associated with any physical effort or local trauma. Spontaneous formation of an extensive cervical hematoma, which evolved in 3 hours with tracheal compression and displacement, which required emergency surgical approach. In the postoperative period, the patient was transferred to intensive therapy, and initiated endovenous replacement of calcium and magnesium. The patient progressed well and did not require new surgical procedures. It was chosen to correct anticoagulation with low molecular weight heparin until the 21^st^ postoperative day, when, and only then, rivaroxaban administration was reinitiated. No dose adjustment is required in patients with mild (creatinine clearance − CrCl ≤80-50mL/minute) or moderate renal impairment (CrCl <50-30mL/minute). Limited clinical studies for patients with severe renal impairment (CrCl <30-15mL/minute) indicate that plasma levels increase significantly in this patient population. Its use is not recommended for patients with CrCl <15mL/minute.^3^

The patient was submitted to extensive workup for coagulation disorders, including C and S proteins, mutations for factor V of Leiden and the prothrombin gene, homocysteine, plasminogen activator inhibitor, activity of factor VIII, resistance test of activated C protein, lupus anticoagulant, anti-thrombin, anti-cardiolipin, anti-thrombin 3, glycoprotein anti-beta2, D-dimers, iron, ferritin, other elements of the total blood count and ordinary coagulation tests. All results were presentedin the limits of normality.

## DISCUSSION

Hyperparathyroidism secondary to chronic kidney disease is characterized by elevated parathormone levels, gland hyperplasia, and bone disease with high calcium turnover. According to the *Sociedade Brasileira de Nefrologia* (SBN), about 16% of patients on dialysis presented with parathormone levels above 600pg/mL, but 31% were on only calcitriol, 2% on paricalcitol, and 4% on cinacalcete. Partial or total parathyroidectomy recommended by SBN in transplanted patients with chronic kidney disease when, after one year of successful transplant, there has been no regression of levels of persistent parathormone associated with persistent hypercalcemia.^4 , 5^

Hypocalcemia as the cause of hypocoagulability has already been well documented, and the ionized calcium levels should be equal to or greater than 0.9mmol/L to preserve hemostatic function. The incidence of hypocalcemia after parathyroidectomy is widespread in literature, reaching 40%, and primarily depends on the extension of the post parathyroidectomy, to the extension and size of the surgical procedure, presence of a tumor or glandular hyperplasia, the execution or not of auto-implant of parathyroid, and of age. Ionized calcium under 1.05mmol/L was demonstrated as a predictor of symptoms in 95% of patients until the second postoperative day, occurring normalization of serum levels in the first week, with supplementation of oral calcium and administration or not of vitamin D. The delicate balance between the functional pro-thrombotic status and the dysfunctional pathophysiology of coagulation in chronic kidney disease make the execution of procedures in patients who had thromboembolic complications even more challenging than in those on oral anticoagulants.^1^

Recent studies demonstrated that patients with hyperparathyroidism and chronic kidney disease show hypercoagulability and hypofibrinolysis, increasing the incidence of thromboembolic phenomena. The tendency to hypercoagulability in chronic kidney disease could be explained by the multifactorial nature of the condition, with reduced endogenous anticoagulants and fibrinolytic activity, increasing levels of pro-coagulant factors by the use of erythropoietin, while the higher risk of bleeding may be due to anemia and uremia. Therefore, there should be constant concern with the possibility of increased bleeding during surgery and the use of thromboprophylaxis, including thrombosis of the arteriovenous fistula.^6^

Lately, the use of rivaroxaban has been extended beyond the procedural indications, because the drug is considered safe, with efficacy comparable to low molecular weight heparin and warfarin, besides not need for laboratory control tests. Its use does not require dose adjustments for weight, age or sex, and it does not present interactions with food, which improves its absorption and bioavailability when taken at meals. The drug half-life is only 5 to 9 hours in adults, providing rapid and short-duration anticoagulation.^1^ Phase 3 clinical trials demonstrated that despite efficacy and safety rates comparable to or even better than heparin and warfarin, the risk of severe bleeding was of 0.7% and 3.6%.^7 - 9^ Nevertheless, cases of bleeding are numerous and recent study carried out in Sweden showed, in a 2-year period, there were 84 cases of severe hemorrhage (70.2% intracranial) associated with the use of rivaroxaban and apixaban. These patients were treated with prothrombin complex; however, in only 69%, hemostasis was adequate, with a total mortality rate of 32%.^10^ Conventional laboratory tests to evaluate coagulation showed little or very little abnormalities, not presenting clinical usefulness to monitor the pharmacological action of the drug.

## CONCLUSION

Since parathyroid excision is still a standard surgical procedure and hypocalcemia is an expected secondary event, physicians should be aware of clinical complaints and monitor serum calcium levels of patients on rivaroxaban use, due to the possible risk of bleeding during the postoperative period.
