# Supplementary material for: Association between hypocalcemia and rivaroxaban in coagulation disorders: a case report
Source: Einstein (Sao Paulo). 2020 Jan 22;18:eRC4819. doi: 10.31744/einstein_journal/2020RC4819 (PMC6980294; doi:10.31744/einstein_journal/2020RC4819)
Supplement: Translation

# Translation: Associação entre hipocalcemia e rivaroxaban em distúrbios de coagulação: relato de caso

**Authors:** Alexandre Mio Pos, Mateus Arruda Aleixo, Ana Paula Drummond-Lage

**Affiliations:** 1 Brasil Faculdade Ciências Médicas de Minas Gerais, Belo Horizonte, MG, Brasil.

**Keywords:** Rivaroxabana/efeitos adversos, Coagulação sanguínea, Hematoma, Paratireoidectomia

## Abstract

Descrevemos um paciente com hiperparatireoidismo terciário com história de três episódios de trombose venosa profunda e em uso de rivaroxabana. O paciente foi submetido a uma paratireoidectomia subtotal, desenvolvendo hematoma cervical com compressão das vias aéreas. Foi necessária descompressão cirúrgica de emergência. No nono dia de pós-operatório, os níveis séricos de cálcio iônico estavam baixos. O conhecimento da equipe médica sobre doenças preexistentes e de sua implicação no estado de coagulação é condição indispensável para a redução da morbimortalidade do procedimento cirúrgico. No entanto, não há relatos na literatura associando hipocalcemia com o uso da nova classe de anticoagulantes que atuam como inibidores do fator X (fator de Stuart-Prower), predispondo ao aumento do sangramento no pós-operatório imediato.

## INTRODUÇÃO

Rivaroxabana é um anticoagulante da nova classe de inibidores seletivos do fator X ativado, bastante usado na prevenção e no tratamento de eventos tromboembólicos, especificamente em pacientes com doenças cardiovasculares.^1^ O emprego da rivaroxabana não exige monitorização da coagulação, pois esse medicamento tem propriedades farmacológicas previsíveis, como meia-vida curta e ação rápida, com perfil de segurança aprimorado, o que resulta no uso de uma dose fixa.^1^ No entanto, em alguns quadros clínicos potencialmente graves, deve-se realizar uma análise da ação da rivaroxabana, mesmo que indiretamente, por meio da avaliação do tempo de protrombina ou da determinação da atividade do antifator Xa.^1^ Entre esses quadros clínicos, destacam-se os pacientes com hiperparatiroidismo ou com doença renal em estágio 4 ou 5, pois constituem um grupo de alto risco para eventos trombóticos. O uso de anticoagulantes nessa categoria de pacientes não é incomum.

Os níveis séricos de anticoagulantes orais dependem diretamente da função renal. Consequentemente, uma redução do *clearance* renal pode afetar a eficácia e a segurança desses medicamentos. Os pacientes com doença renal crônica têm sua própria curva farmacocinética acima do limite de normalidade, o que deve ser levado em consideração ao se analisar o risco de hemorragia.^1 , 2^ Devido a esses fatores, a conduta clínica no tratamento com anticoagulantes em pacientes com doença renal crônica é um desafio médico.

Um termo de consentimento informado por escrito foi obtido do paciente.

## RELATO DE CASO

Apresentamos um paciente de 51 anos, sexo masculino, com história de doença renal crônica em estágio 5, de etiologia indeterminada, em diálise desde 2003, com transplante intervivos e HLA/ABO-incompatível em 2014. Como complicação da patologia, o paciente apresentou dislipidemia, hiperuricemia, hipovitaminose D (17ng/mL), osteoporose com T-score de -5,9, calcificação extraesquelética com aterosclerose avançada, três eventos tromboembólicos venosos em membro inferior e hiperparatireoidismo terciário. Foi iniciado uso contínuo de rivaroxabana, na dose de 20mg ao dia, e de cinacalcete, na dose de 30mg ao dia. Como o paciente manteve intensa atividade osteoclástica, com níveis de paratormônio acima de 300pg/mL e cálcio iônico constantemente acima do limite máximo da normalidade (1,60mg/dL) 2 anos após o transplante, e com função renal já normalizada (creatinina 1,10mg/dL), a equipe decidiu pela excisão cirúrgica parcial das paratireoides. A rivaroxabana foi suspensa 48 horas antes do procedimento cirúrgico, e o antifator Xa foi dosado (0,004UI/mL). Depois disso, foi iniciada a transição da anticoagulação com heparina de baixo peso molecular por 6 dias. O procedimento foi realizado sem anormalidades e com critérios laboratoriais de excisão efetiva (paratormônio perioperatório 8pg/mL). Na terapia intensiva, o paciente evoluiu apenas com hipocalcemia assintomática, controlada com cálcio oral. No quinto dia pós-operatório, foi reiniciada a rivaroxabana 20mg ao dia, sem dose de ataque, seguindo-se a alta hospitalar. O paciente evoluiu bem. No entanto, tardiamente, no nono dia pós-operatório, o paciente apresentava cálcio iônico de 0,79mmol/L, apesar da reposição oral de cálcio (4,5g ao dia), calcitriol (2,25mg ao dia) e magnésio (3g ao dia), quando surgiram desconforto e dor incisional súbita, não associados a esforço físico nem à trauma local. Houve formação espontânea de extenso hematoma cervical, que evoluiu em 3 horas para compressão e desvio da traqueia, sendo necessária abordagem cirúrgica de emergência. No pós-operatório, o paciente foi transferido para a terapia intensiva, sendo iniciada a reposição endovenosa de cálcio e magnésio. A evolução do paciente foi boa, não exigindo novos procedimentos cirúrgicos. Foi escolhida correção da anticoagulação com heparina de baixo peso molecular até o 21^o^dia pós-operatório, quando, e somente então, foi reiniciada a administração da rivaroxabana. Nenhum ajuste de dose é necessário em pacientes com disfunção renal leve ( *clearance* de creatinina − ClCr≤80-50 mL/minuto) ou moderada (ClCr <50-30mL/minuto). Alguns estudos clínicos de pacientes com insuficiência renal grave (ClCr <30-15 mL/minuto) indicam que os níveis plasmáticos aumentam significativamente nessa população de pacientes. Seu uso não é recomendado em pacientes com ClCr <15mL/minto.^3^

O paciente foi submetido a inúmeros exames propedêuticos para pesquisa de distúrbios de coagulação, incluindo proteínas C e S, mutações do gene da protrombina e do fator V de Leiden, homocisteína, inibidor do ativador do plasminogênio, atividade do fator VIII, teste de resistência da proteína C ativada, anticoagulante lúpico, antitrombina, anticardiolipina, antitrombina 3, antibeta-2-glicoproteína, dímero D, ferro, ferritina, outros elementos do hemograma total e exames comuns de coagulação. Todos os resultados se mostraram nos limites da normalidade.

## DISCUSSÃO

O hiperparatireodismo secundário à doença renal crônica caracteriza-se pela elevação dos níveis de paratormônio, hiperplasia glandular e doença óssea com alto *turnover* de cálcio. De acordo com a Sociedade Brasileira de Nefrologia (SBN), cerca de 16% dos pacientes em diálise apresentam nível de paratormônio superior a 600pg/mL, mas 31% recebiam apenas calcitriol, 2% recebiam paracalcitol, e 4% recebiam cinacalcete. A paratireodectomia parcial ou total é recomendada pela SBN em pacientes pós-transplantados com doença renal crônica quando, após 1 ano de um transplante bem-sucedido, não tenha havido regressão dos níveis persistentes de paratormônio associados à hipercalcemia persistente.^4 , 5^

A hipocalcemia como causa de hipocoagulabilidade já está bem documentada, e o nível de cálcio iônico deve ser ≥0,9mmol/L para preservar a função hemostática. A incidência de hipocalcemia pós-paratireoidectomia é ampla na literatura, chegando a 40%, e depende principalmente da extensão do período pós-paratireoidectomia, da extensão e tamanho do procedimento cirúrgico, da presença de um tumor ou hiperplasia glandular, da execução ou não de autoimplante da paratireoide, e da idade. Foi demonstrado que cálcio iônico abaixo de 1,05mmol/L é preditor de sintomatologia em 95% dos pacientes até o segundo dia pós-operatório, ocorrendo a normalização dos níveis séricos na primeira semana, com suplementação de cálcio oral e administração ou não de vitamina D. O delicado equilíbrio entre a condição pró-trombótica funcional e a fisiopatologia disfuncional da coagulação na doença renal crônica tornam a execução de procedimentos em pacientes que já tiveram complicações tromboembólicas ainda mais desafiadora do que nos que estão recebendo anticoagulantes orais.^1^

Estudos recentes demonstraram que pacientes com hiperparatireoidismo e doença renal crônical apresentam hipercoagulabilidade e hipofibrinólise, aumentando a incidência de fenômenos tromboembólicos. A tendência à hipercoagulabilidade na doença renal crônica poderia ser explicada pela natureza multifatorial da enfermidade, com redução dos anticoagulantes endógenos e da atividade fibrinolítica, aumentando os níveis de fatores pró-coagulantes pelo uso de eritropoietina, ao passo que o maior risco de sangramento pode ser por anemia e uremia. Portanto, a preocupação com a possibilidade de maior sangramento cirúrgico e o uso de tromboprofilaxia, incluindo a trombose da própria fístula arteriovenosa, deve ser uma constante na abordagem cirúrgica.^6^

Recentemente, o uso da rivaroxabana foi estendido para além das indicações do procedimento, porque o medicamento é considerado seguro, com eficácia comparável à da heparina de baixo peso molecular e da varfarina, além de não necessitar de exames laboratoriais de controle. Seu uso não necessita de ajustes de dose para peso, idade ou sexo, e não apresenta interações com alimentos, o que melhora ainda mais sua absorção e biodisponibilidade quando administrado nas refeições. O medicamento tem meia-vida de apenas 5 a 9 horas em adultos, proporcionando anticoagulação rápida e de curta duração.^1^ Ensaios clínicos de fase 3 demonstraram que, apesar das taxas de eficácia e segurança comparáveis ou até melhores do que as de heparina e varfarina, o risco de sangramento grave foi de 0,7% e 3,6%.^7 - 9^ Não obstante, os casos de sangramento são numerosos, e um estudo recente realizado na Suécia mostrou que, em um período de 2 anos, houve 84 casos de sangramento grave (70,2% intracranianos) associados ao uso da rivaroxabana e da apixabana. Esses pacientes foram tratados com complexo de protrombina, contudo, em apenas 69% dos casos, a hemostasia foi adequada, com taxa de mortalidade total de 32%.^10^ Os exames laboratoriais convencionais para avaliação da coagulação mostravam alterações pequenas ou mínimas, não tendo utilidade clínica para o acompanhamento da ação farmacológica do medicamento.

## CONCLUSÃO

Como a excisão da paratireoide ainda é um procedimento cirúrgico padrão, e a hipocalcemia é um evento secundário esperado, os médicos devem manter-se atentos a queixas clínicas e monitorar o cálcio sérico de pacientes em uso de rivaroxabana, devido ao possível risco de hemorragia no período pós-operatório.
